# Calcineurin/NFATc3 pathway mediates myocardial fibrosis in diabetes by impairing enhancer of zeste homolog 2 of cardiac fibroblasts

**DOI:** 10.1186/s12872-023-03492-5

**Published:** 2023-09-21

**Authors:** Lei Zhang, Huan-Huan Liu, Fan Yang, Zhi-Yuan Zhang, Ying Wu, Feng Li, Shi-Peng Dang, Zhen-Ye Zhang, Ling-Ling Qian, Ru-Xing Wang

**Affiliations:** 1 Department of Cardiology, Wuxi People’s Hospital Affiliated to Nanjing Medical University, Wuxi, 214023 China; 2 Wuxi School of Medicine, Jiangnan University, Wuxi, 214122 China

**Keywords:** Diabetes, Myocardial fibrosis, Calcineurin, Nuclear factor of activated T cell, Enhancer of zeste homolog 2

## Abstract

**Background:**

Diabetes is associated with myocardial fibrosis, while the underlying mechanisms remain elusive. The aim of this study is to investigate the underlying role of calcineurin/nuclear factor of activated T cell 3 (CaN/NFATc3) pathway and the Enhancer of zeste homolog 2 (EZH2) in diabetes-related myocardial fibrosis.

**Methods:**

Streptozotocin (STZ)-injected diabetic rats were randomized to two groups: the controlled glucose (Con) group and the diabetes mellitus (DM) group. Eight weeks later, transthoracic echocardiography was used for cardiac function evaluation, and myocardial fibrosis was visualized by Masson trichrome staining. The primary neonatal rat cardiac fibroblasts were cultured with high-glucose medium with or without cyclosporine A or GSK126. The expression of proteins involved in the pathway was examined by western blotting. The nuclear translocation of target proteins was assessed by immunofluorescence.

**Results:**

The results indicated that high glucose treatment increased the expression of CaN, NFATc3, EZH2 and trimethylates lysine 27 on histone 3 (H3K27me3) in vitro and in vivo. The inhibition of the CaN/NFATc3 pathway alleviated myocardial fibrosis. Notably, inhibition of CaN can inhibit the nuclear translocation of NFATc3, and the expression of EZH2 and H3K27me3 protein induced by high glucose. Moreover, treatment with GSK126 also ameliorated myocardial fibrosis.

**Conclusion:**

Diabetes can possibly promote myocardial fibrosis by activating of CaN/NFATc3/EZH2 pathway.

**Supplementary Information:**

The online version contains supplementary material available at 10.1186/s12872-023-03492-5.

## Background

Diabetes mellitus (DM) is a complex metabolic disorder and DM-related cardiovascular complications are the leading cause of death among DM patients [1, 2]. Myocardial fibrosis is a common and prominent feature of diabetic heart disease, culminating in heart failure or sudden death [3]. However, how DM is responsible for myocardial fibrosis remains unknown. Despite this, no evidence-based therapies are explicitly directed at alleviating myocardial fibrosis.

Calcineurin (CaN), a Ca^2+^/calmodulin-dependent serine/threonine phosphatase, functions by dephosphorylating target proteins in eukaryotes [4]. Nuclear factor of activated T cells 3 (NFATc3), as one of the substrates of CaN, can be dephosphorylated and enter the nucleus [5]. Several studies have indicated that the CaN/NFATc3 pathway was intimately related to myocardial fibrosis in spontaneously hypertensive rats [6] and angiotensin II induced cardiac hypertrophy [7]. Moreover, high glucose was reported to activate the CaN/NFAT pathway in H9C2 cells [8, 9]. However, studies on the role of this pathway in diabetes-induced myocardial fibrosis are limited.

Enhancer of zeste homolog 2(EZH2), an enzymatic member of polycomb repressive complex 2, is responsible for catalyzing trimethylation of histone 3 lysine 27 (H3K27me3) [10]. Recent evidence suggested that EZH2 is associated with atrial fibrosis [11]. A previous study demonstrated that EZH2 was overexpressed in diabetic cardiomyopathy mice and high glucose cultured mouse cardiomyocytes [12]. Furthermore, NFAT can bind to the EZH2 promoter [13]. Nevertheless, the role of the CaN/NFATc3 pathway and whether EZH2 may be regulated by the CaN/NFATc3 pathway in diabetes-induced myocardial fibrosis is not fully understood.

This study aimed to investigate the effects of the CaN/NFATc3 pathway in DM-related myocardial fibrosis and to clarify the relationship between the CaN/NFATc3 pathway and EZH2 in DM-related myocardial fibrosis in vitro and in vivo.

## Methods

### Experimental animals

Male Sprague-Dawley (SD) rats (6–8 weeks, 150–200 g, n = 40) were purchased from Changzhou Cavens Laboratory Animal Company in China. The rats were kept in animal rooms and were reared under a 12 h light/12 h dark cycle with controlled temperature (23 ± 1℃). One week after adaptive feeding, SD rats were randomly assigned into two groups (n = 8 for each group): the diabetes mellitus (DM) group and the control (Con) group. A rat model of diabetes mellitus was established following a previously reported protocol [14]. Briefly, seven days after being intraperitoneally injected with streptozotocin (STZ, Sigma-Aldrich, S0130, 60 mg/kg), blood glucose levels greater than 16.7 mmol/L were included in this study. Measurements of blood glucose and body weight were performed weekly. Eight weeks later, rats were anesthetized by intraperitoneal injection of sodium pentobarbital (60 mg/kg), and the hearts were quickly moved and stored at -80℃. All animal experiments complied with the Guide for the Care and Use of Laboratory Animals (National Institutes of Health Publication No. 85 − 23, revised in 1996) and were reported following the Animal Research Report of In Vivo Experiments (ARRIVE) guidelines.

### Echocardiography evaluation

Echocardiography examination was performed on five mice per group. On the setting of 2% isoflurane anesthesia, we used echocardiography (Philip, ie33) to evaluate cardiac function. Derived echocardiography parameters including left ventricular ejection fraction (EF), left ventricular fractional shortening (FS), left ventricular internal diameter at end-diastole (LVIDd), and left ventricular internal diameter at end-systole (LVIDs) were documented. All measurements were performed by an observer blinded to the tracings’ identity.

### Histopathological analysis

The heart was fixed in 4% paraformaldehyde, embedded in paraffin, and cut into 4 μm thick slices. Hematoxylin and eosin (HE, Beyotime, C0105) staining were used for histological analysis. We further examined myocardial fibrosis by Masson trichrome staining (Nanjing Jiancheng Bioengineering Institute, D026).

### Primary culture of neonatal rat cardiac fibroblasts

Primary neonatal rat cardiac fibroblasts (NRCFs) were isolated and cultured according to the method in the previously reported literature [14]. In brief, 1–3 days old neonatal rats were disinfected with 75% ethanol and the hearts were removed from the thorax and then washed the heart three times repeatedly with pre-cooled phosphate buffered saline (PBS, Gibco, 10010-023). Then ventricles were minced and digested with 0.125% trypsin (Gibco, 25200072) and 0.1% collagenase (Worthington, LS004176). The cell suspension was obtained from the digested tissue. Digestion was terminated with DMEM containing 10% fetal bovine serum (FBS, Gibco, 12664025) and then transferred to 100 mm culture dishes (Corning, 430167). After an hour cultured in a 37℃, 5.0% CO_2_ incubator, new culture media were added after removing the supernatant.

### Hyperglycemia cell model and treatments

Primary neonatal rat Cardiac Fibroblasts were divided into two groups: the normal glucose (Con) group and the high glucose (HG) group. In the Con group, NRCFs were cultured in the low-glucose condition of 5.5 mmol/L, and HG group cells were cultured in the high-glucose concentration medium with 25 mmol/L glucose. To investigate the role of CaN/NFATc3/EZH2 in myocardial fibrosis due to high glucose, we used 10 µmol/L cyclosporin A (CsA, a CaN inhibitor, Medchemexpress, HY15465) [15], or 1 µmol/L GSK126 (an EZH2 inhibitor, Medchemexpress, HY13470) to treat cells for 72 h. Cells were harvested after 72 h.

### Western blot analysis

Rat heart tissues were homogenized for tissue lysate extraction in lysis buffer with a cocktail of protease inhibitors, and cell lysates were centrifuged for 15 min. Proteins were then transferred to PVDF membranes, which were placed in the blocking solution (Beyotime, P0252) at room temperature for 15 min. Then, the membrane was incubated overnight at 4 °C with primary antibodies. The primary antibodies for CaN (Santa Cruz, SC-117808), NTATc3 (Santa Cruz, SC-8405), EZH2 (Cell Signaling Technology, 5246S), H3K27me3 (Cell Signaling Technology, 9733S), H3 (Cell Signaling Technology, 14269S), TGF-β1 (Abcam, ab179695), Collagen I (Proteintech, 14695-1-AP), Collagen III (Proteintech, 22734-1-AP) and β-actin (Abcam, ab6276) were used. Next, secondary antibody was added and incubated at room temperature. Bands were then quantified using Image J.

### Immunofluorescence analysis

For the nuclear translocation of NFATc3, NRCFs cells were divided the cells into four groups: (1) control group: NRCF cells were cultured in normal glucose; (2) control + CSA group: NRCF cells were pretreated with CsA (10 µmol/L) in normal glucose; (3) HG group: NRCF cells were incubated with 25 mmol/L glucose; (4) HG + CsA group: cells were given 25 mmol/L glucose and CsA. After 72 h, the cells were washed with PBS and fixed by 4% paraformaldehyde (Servicebio, G1101), and then washed again with PBS. After that, cells were permeated with 0.5% Triton X-100 (Sigma-Aldrich, T9284) at room temperature for 15 min and washed thrice with PBS for 3 min each. After that, cells were blocked with normal goat serum (Solarbio, SL038) for 30 min. Primary antibodies were diluted with 1% BSA. Cells were plated at 4 °C for 24 h and then washed thrice with PBS for 3 min each. Secondary antibody diluted with 1% BSA in PBS was added and incubated for one hour. Next, secondary antibody was removed and cells were washed thrice with PBS for 5 min each. Cells were incubated with DAPI (Beyotime, C1005) for 5 min at room temperature and then were performed by three washes with PBS. Later, cells were observed using a fluorescence microscope (ThermoFisher, EVOS).

### Statistical analysis

All data were expressed as the mean ± standard error. Each group of data was performed to normality and homogeneity of variance testing. Statistical comparisons were completed using One-way ANOVA or t-test with SPSS 26.0. *P* < 0.05 was considered statistically significant.

## Results

### Hyperglycemia aggravated the systolic dysfunction

**Fig. 1:**
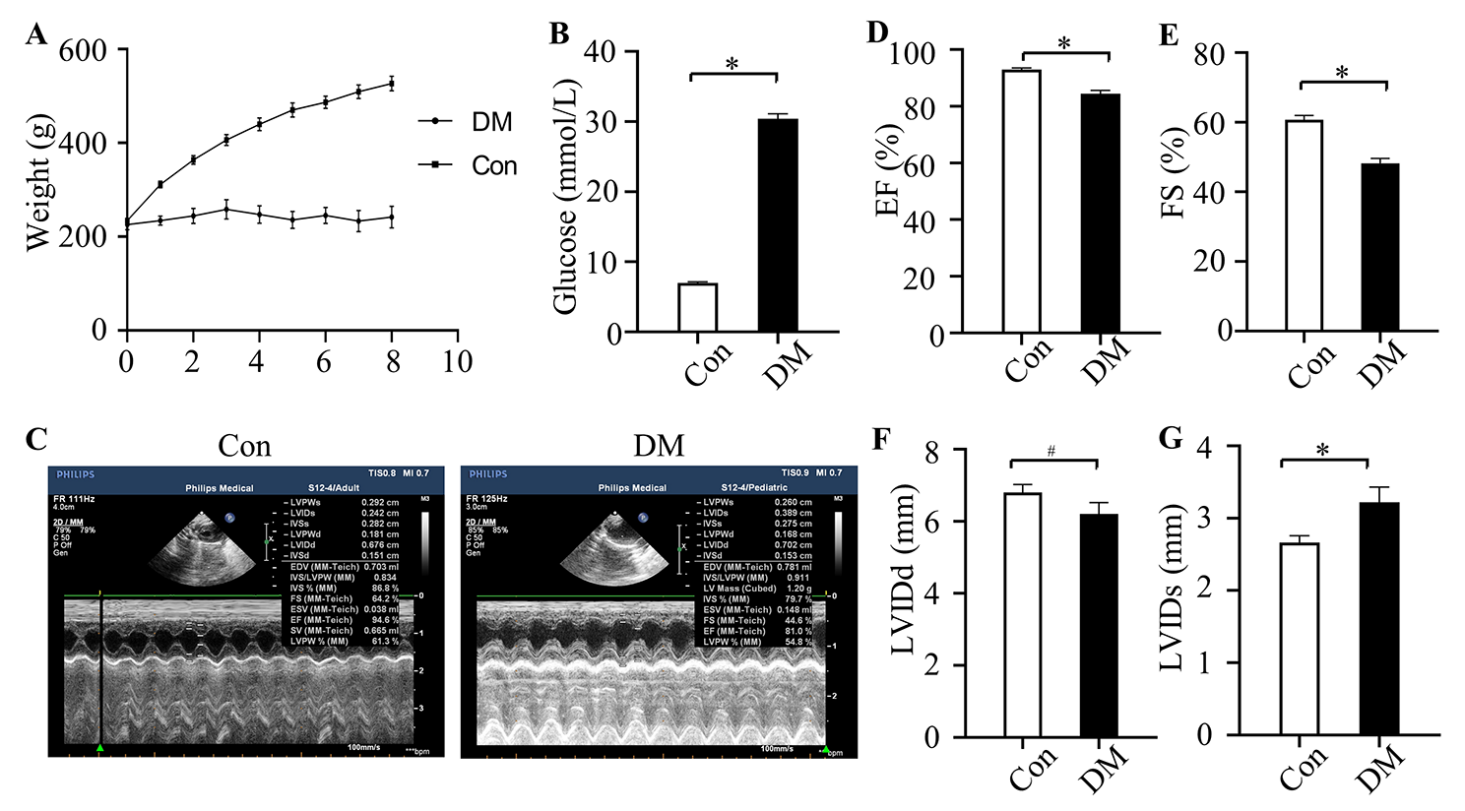
The cardiac function in diabetic rats. (**A**) The body weight over 8 weeks in Con and DM groups. (**B**) The blood glucose over 8 weeks in different groups. (**C-G**) The analysis of left ventricular EF, FS, LVIDd and LVIDs in different groups. Con: control group; DM: diabetes mellitus group; FS: left ventricular fractional shortening; EF: ejection fraction; LVIDd: left ventricular internal diameter at end-diastole; LVIDs: left ventricular end systolic diameter. n = 5 per group, *P < 0.05

Compared to the Con group, the DM group had elevated blood glucose levels, whereas the body weight of the rats in the DM group was substantially lower (Fig. 1A and B). We used echocardiography to assess cardiac functions (Table 1). Representative echocardiogram images in Con and DM groups were shown in Fig. 1C. The results revealed that EF, FS and LVIDd were greatly reduced in the DM group compared to the Con group (Fig. 1D-F). Meanwhile, there was a significantly increase in LVIDs in DM groups (Fig. 1G). The results showed that the cardiac systolic function was impaired in diabetes.

**Table 1:** Echocardiographic analysis in different groups

	Con	DM	*P*
LVEF(%)	92.33 ± 1.50	84.43 ± 2.95	0.00
FS(%)	60.77 ± 2.97	48.25 ± 3.43	0.00
LVIDd(cm)	6.80 ± 0.54	6.21 ± 0.77	0.15
LVIDs(cm)	2.66 ± 0.23	3.22 ± 0.52	0.36
LVPWd(cm)	0.19 ± 0.29	0.16 ± 0.27	0.15
LVPWs(cm)	0.31 ± 0.34	0.28 ± 0.38	0.13
LVSd(cm)	0.16 ± 0.19	0.14 ± 0.19	0.15
LVSs(cm)	0.27 ± 0.33	0.23 ± 0.31	0.38
EDV(ml)	0.72 ± 0.16	0.57 ± 0.18	0.16

n = 6 for each group. LVEF, left ventricular ejection fraction; FS, fractional shortening; LVIDd, left ventricular internal dimension at end diastole; LVIDs, left ventricular internal dimension at end systole; LVPWd, left ventricular posterior wall thickness at end diastole; LVPWs, left ventricular posterior wall thickness at end systole; IVSd, interventricular septal thickness at end diastole; IVSs, interventricular septal thickness at end systole; EDV, end-diastolic volume.

### Hyperglycemia contributed to myocardial fibrosis

**Fig. 2:**
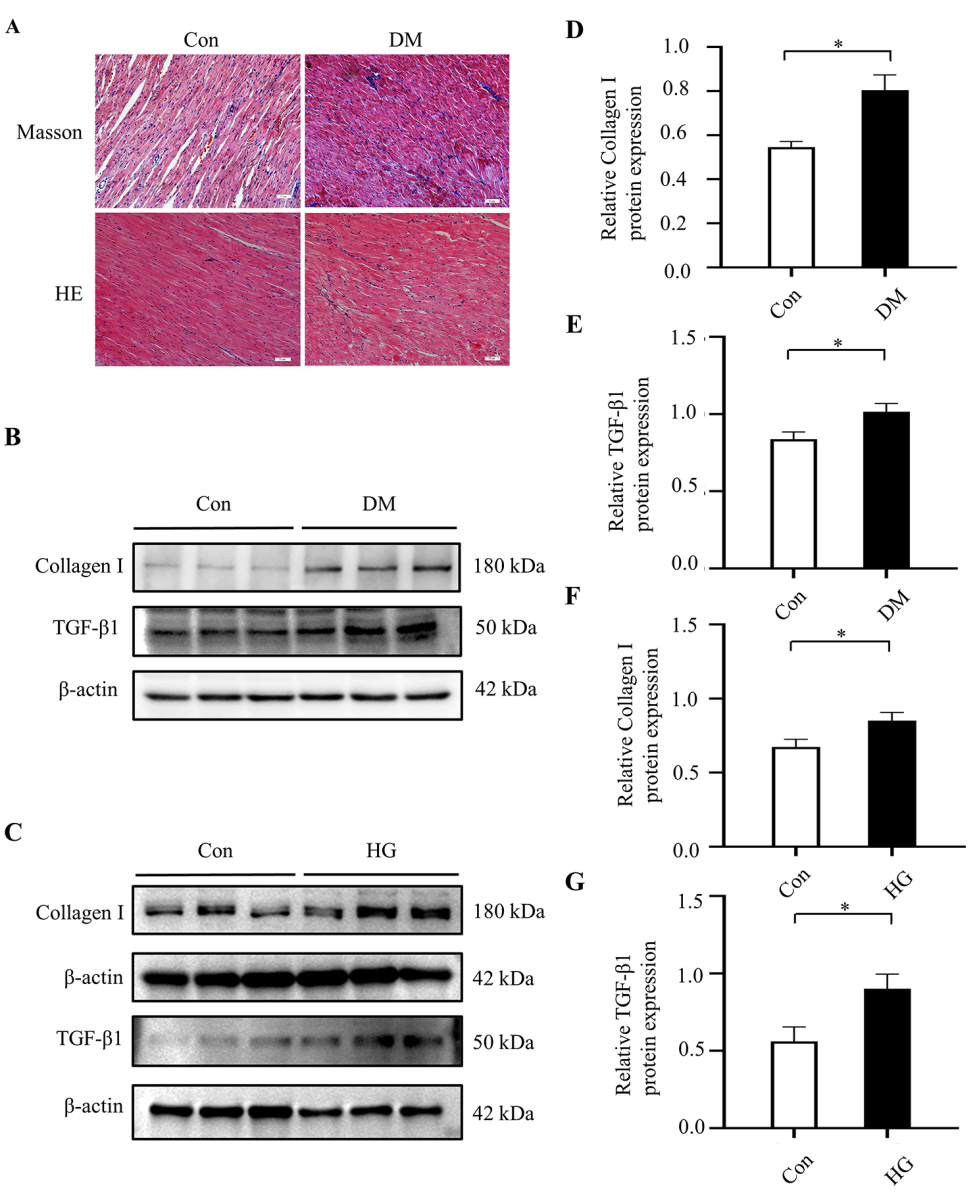
Hyperglycemia promoted myocardial fibrosis. (**A**) Representative images of Masson and hematoxylin and eosin (HE) staining for myocardial tissue in the Con and DM groups (n = 3 per group). (**B, D, E**) The protein expressions of Collagen I, Collagen III and TGF-β1 and in rat hearts of the two groups (n = 6 per group). (**C, F, G**) The protein expressions of TGF-β1 and collagen I in NG and HG groups (n = 6 per group). The blots in Fig. 2B were from different parts of the same gel. The blots in Fig. 2C were from different gels. Uncropped protein blots are provided in the supplementary file. Con: control group; DM: diabetes mellitus group; HG: high glucose group. *P < 0.05

Morphological changes in left ventricular tissue were visualized by HE and Masson staining (Fig. 2A). HE staining showed that myocardial fibers were arranged in a disordered manner significantly, and the myocardial tissues exhibited fractures and bends in the DM group compared with the Con group. Masson staining revealed severe fibrosis of the ventricular area in the DM group. Expressions of Collagen I and TGF-β1 proteins were significantly increased in the ventricular tissue of diabetic rats (Fig. 2B-D), which was consistent with the results obtained in NRCFs cultured in high glucose (Fig. 2E-G). Taken together, the above results suggested that diabetes could induce myocardial fibrosis.

### Hyperglycemia promoted the activation of CaN/NFATc3 pathway

**Fig. 3:**
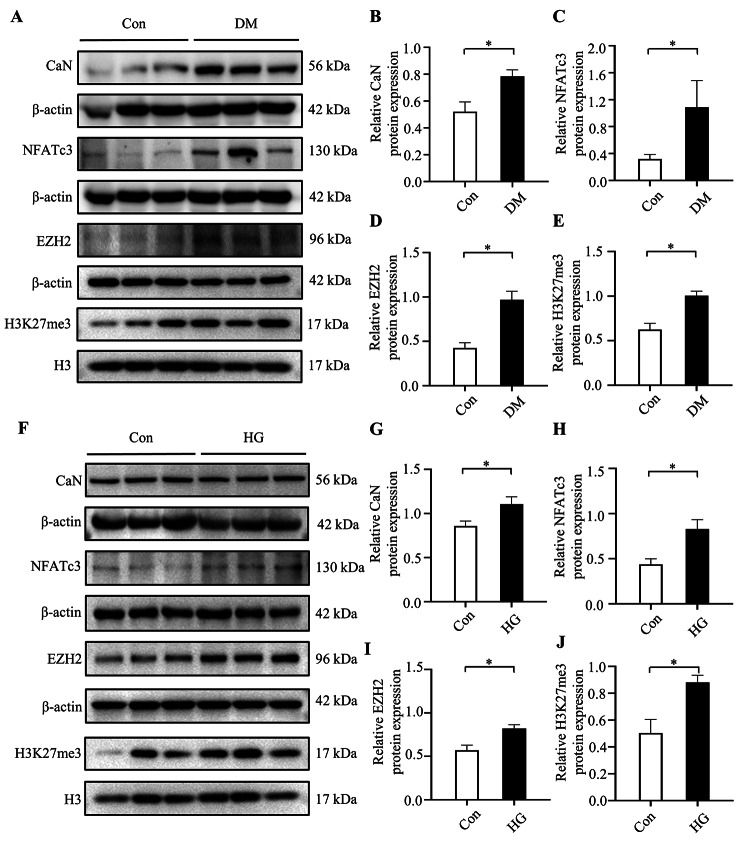
Hyperglycemia promoted the expression of CaN/NFATc3 pathway and EZH2 protein. (**A-E**) The protein expressions of CaN, NFATc3, EZH2 and H3K27me3 in rat hearts of the two groups (n = 6 per group). (**F-J**) The protein expressions of CaN, NFATc3, EZH2 and H3K27me3 in NRCFs of the two groups (n = 6 per group). The blots in Fig. 3 were from different gels. Con: control group; DM: diabetes mellitus group; HG: high glucose group. *P < 0.05

To determine the potential mechanisms by which hyperglycemia promotes myocardial fibrosis, we conducted the expression of CaN/NFATc3 pathway and a potential downstream protein of this signaling, EZH2, in the diabetic heart tissues and NRCFs exposed to high glucose for 72 h. Compared with Con group, the expression of CaN and NFATc3 protein was significantly upregulated in the DM group and HG group respectively (Fig. 3A-C, F-H). The EZH2 protein and its downstream target, H3K27me3 also increased in the two groups (Fig. 3A, D-F, I-J). These results suggested that hyperglycemia activated the CaN/NFATc3 pathway and increased the expression of EZH2. Meanwhile, H3K27me3 was significantly increased by upregulated EZH2 expression.

### Effect of CaN/NFATc3 pathway activation in myocardial fibrosis

**Fig. 4:**
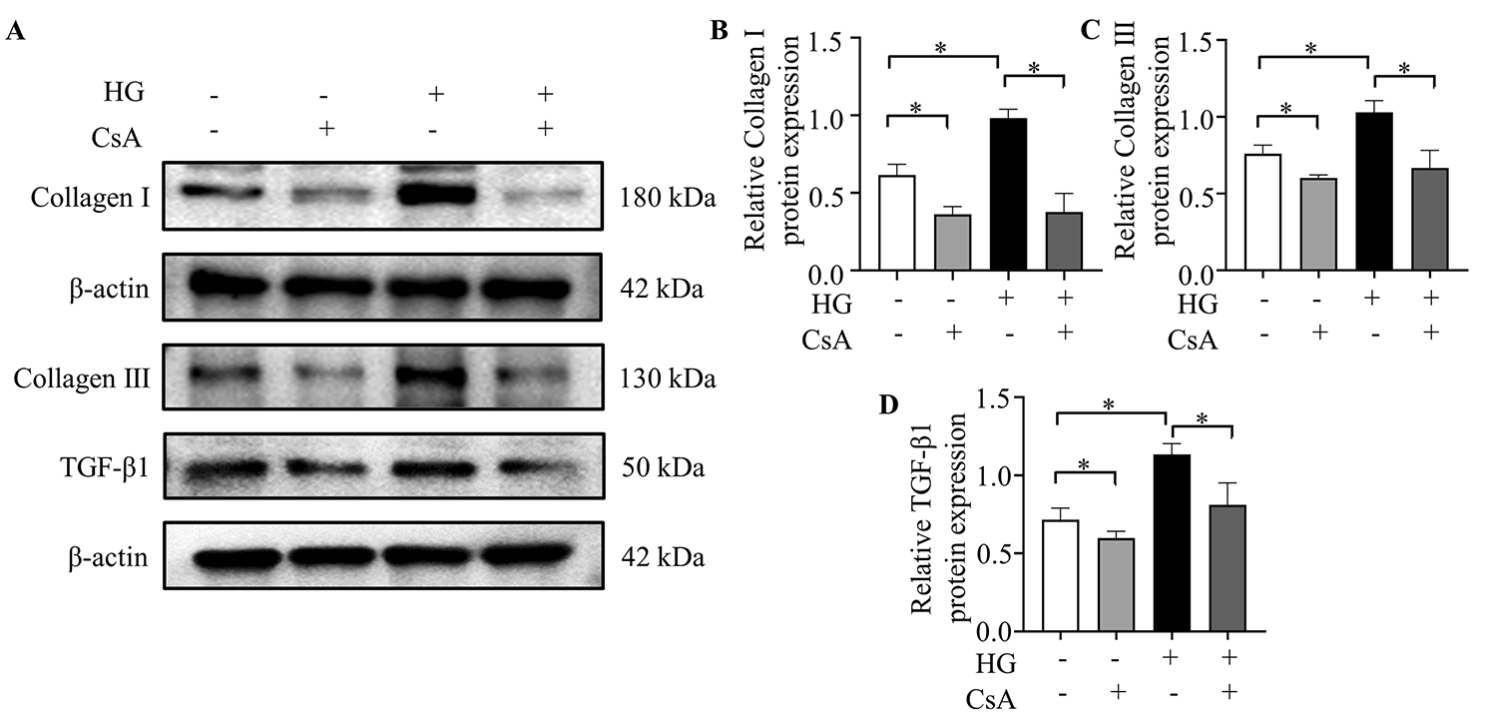
Role of CaN/NFATc3 pathway in hyperglycemia-induced myocardial fibrosis. (**A**) Representative western blotting bands of Collagen I, Collagen III and TGF-β1; (**B-D**) Analysis of the expression of Collagen I, Collagen III and TGF-β1 among different groups. The blots for Collagen III and TGF-β1 in Fig. 4A were from different parts in the same gels; The blots for Collagen I were from different gels. Con: normal glucose group; Con + CsA: normal glucose group with CsA; DM: high glucose group; DM + CsA: high glucose group with CsA. n = 5 per group, *P < 0.05

To probe the role of CaN/NFATc3 pathway in myocardial fibrosis, a CaN-inhibitor, CsA was added to the NRCFs. As shown in Fig. 4, the fibrosis-related proteins, Collagen I, Collagen III and TGF-β1 were markedly downregulated after CsA treatment. Postmortem analysis revealed the treatment alleviated myocardial fibrosis significantly.

### Hyperglycemia promoted CaN/NFATc3 pathway-induced myocardial fibrosis via activating EZH2

**Fig. 5:**
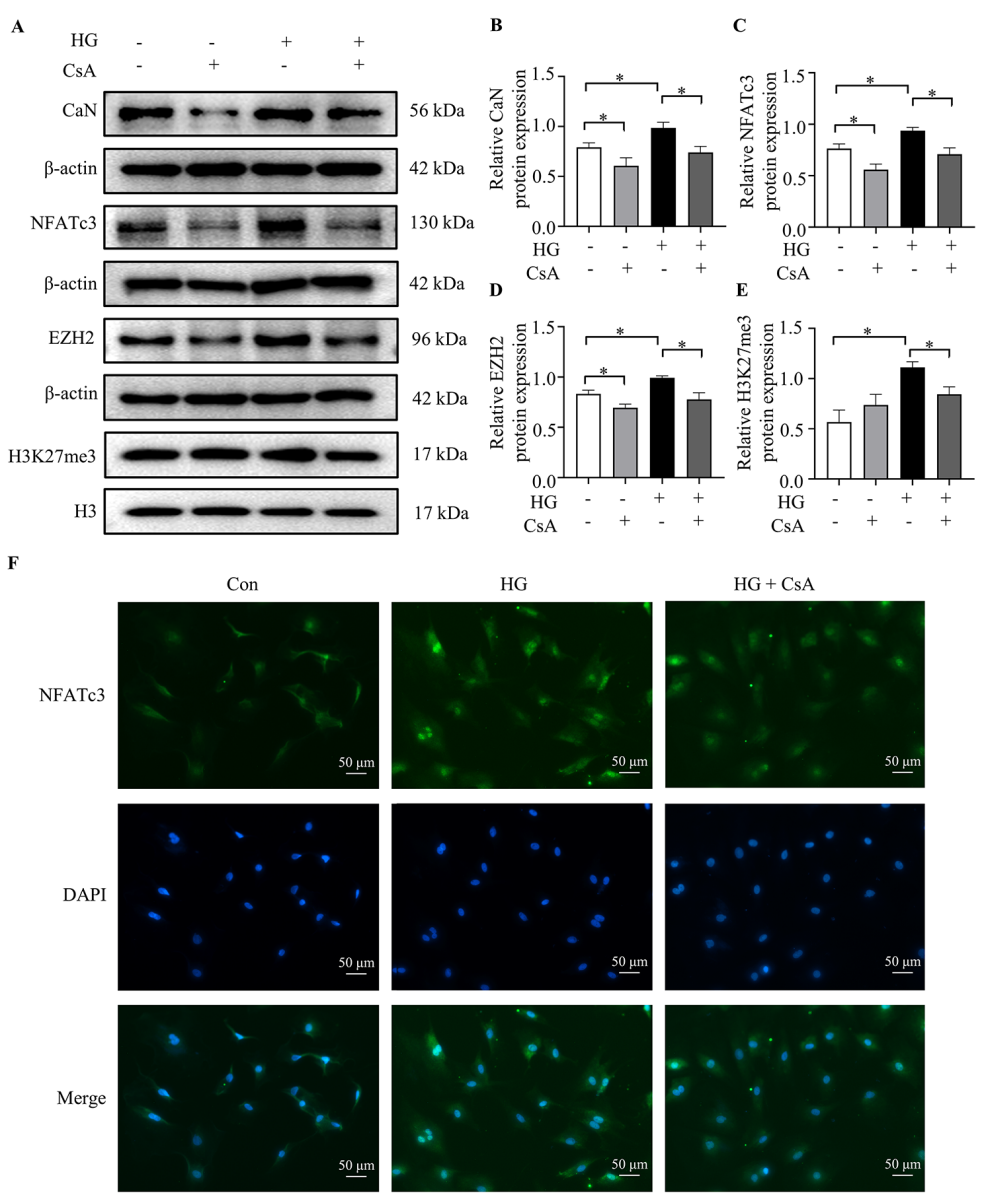
Role of CaN/NFATc3 on EZH2 in hyperglycemia. (**A-B**) the expression of CaN protein levels after use of CsA (n = 5 per group); (**C**) the expression of NFATc3 protein levels after the use of CaN inhibitors (n = 5 per group); (**D**) the expression of EZH2 protein expression after using of CsA (n = 5 per group); (**E**) the expression of H3K27me3 protein expression after using of CsA (n = 5 per group); (**F**) the nuclear translocation of NFATc3. The NRCFs were labeled with anti-NFATc3 (green) and DAPI (blue) (n = 3 per group). The blots in Fig. 5A were from different gels. Con: normal glucose group; Con + CsA: normal glucose group with CsA; DM: high glucose group; DM + CsA: high glucose group with CsA. *P < 0.05

**Fig. 6:**
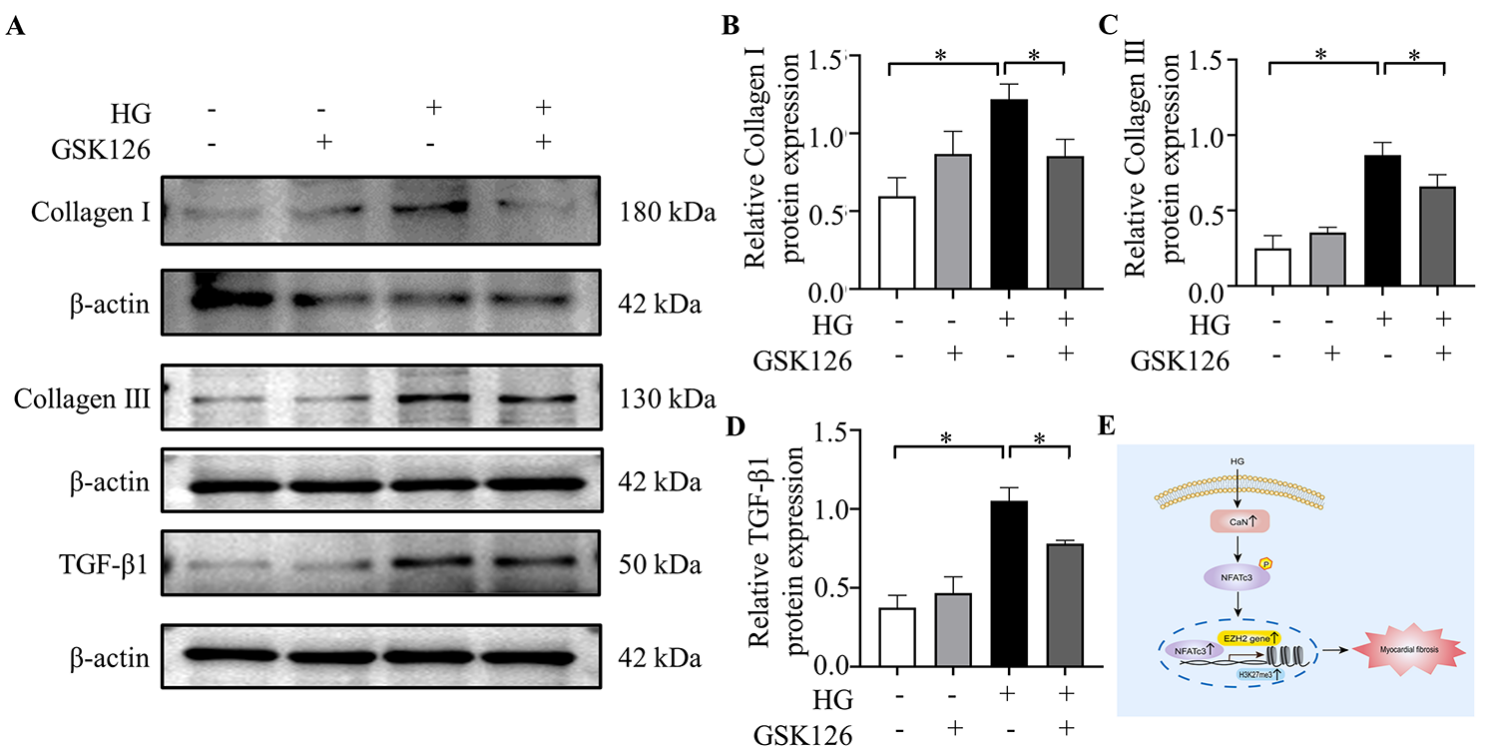
Role of EZH2 in hyperglycemia-mediated myocardial fibrosis in vitro. (**A**) Representative Western blotting bands of Collagen I, Collagen III and TGF-β1 after using GSK126; (**B-D**) Analysis of the expression of Collagen I, Collagen III and TGF-β1 among different groups. (**E**) Schematic depicting the role of CaN/NFATc3/EZH2 pathway in diabetes-induced myocardial fibrosis. The blots in Fig. 6A were from different gels. Con: normal glucose group; Con + EZH2: normal glucose group with EZH2; DM: high glucose group; DM + EZH2: high glucose group with EZH2. n = 3–5 per group, *P < 0.05

To interrogate whether EZH2 is a potential downstream target of CaN/NFATc3 pathway, we found the downregulation of CaN protein levels in Fig. 5A-B after the use of CaN inhibitors. As shown in Fig. 5A, C and F, the expression and the nuclear translocation of the downstream protein, NFATc3 were attenuated. Moreover, EZH2 and H3K27me3 protein expression was significantly recuperated after CsA treatment (Fig. 5D-E). Meanwhile, there was a significant reduction in myocardial fibrosis indicators after GSK126 treatment (Fig. 6A-D). These data indicated that the activation of the CaN/NFATc3 pathway was responsible for the upregulated of EZH2 in cardiomyocytes after exposure to hyperglycemia.

## Discussion

Many studies have shown that myocardial fibrosis contributes to myocardial remodeling and heart failure in diabetes [16–18]. However, the specific mechanisms remain unclear. Thus, the present study investigated whether CaN/NFATc3/EZH2 pathway plays a role in diabetic myocardial fibrosis, thereby presenting a new direction for prevention and treatment of myocardial fibrosis of diabetic patients.

The main findings of this study are: (1) the degree of myocardial fibrosis in the diabetic model rats was significantly increased; (2) CaN/NFATc3 pathway, EZH2 and its downstream target, H3K27me3 were both activated by hyperglycemia; (3) suppressing the expression of CaN and EZH2 can attenuate the enhanced myocardial fibrosis in diabetic rats (Fig. 6E). Based on those findings, we demonstrated that CaN/NFATc3/EZH2 pathway may contribute to the process of myocardial fibrosis induced by DM, suggesting that pharmacological inhibition of CaN/NFATc3/EZH2 pathway may become an effective therapeutic strategy against myocardial fibrosis in diabetes.

Diabetes is a chronic progressive metabolic disease resulting from insufficient insulin production/secretion or insulin resistance [19, 20]. It has been proved that DM can promote myocardial fibrosis and disturb myocardial structure, which, in turn, contributes to impaired myocardial function and heart failure [21]. It has been reported that diabetic mice displayed exaggerated reactive fibrosis, along with increased cardiac oxidative stress and DNA damage [22]. Consistent with previous studies, the expression of fibrosis-related proteins such as Collagen I, Collagen III, TGF-β1 were dramatically increased in high glucose cultured NRCFs and the diabetic rats in the present study. Nonetheless, the concrete mechanism has not been completely understood.

CaN, a serine/threonine protein phosphatase, can be activated by the upstream Ca^2+^-calmodulin complex, and then dephosphorylates the p-NFAT protein, promoting cytoplasmic NFAT subunits to translocate into the nucleus, where they stimulate the transcription of various genes [23]. NFAT protein family is the main substrate of CaN and includes NFAT1 (NFATc2), NFAT2 (NFATc1), NFAT3 (NFATc4), NFAT4 (NFATc3) and NFAT5, among which NFATc3 protein is highly related to the development of cardiovascular disease [7]. Zheng et al. [24] found that stachydrine hydrochloride could inhibit pathological myocardial hypertrophy through the CaN/NFATc3 pathway. The CaN/NFAT pathway has also been revealed to participate in the development of atrial fibrillation in patients with valvular heart disease and diabetes [25]. Beyond those, however, whether the CaN/NFAT pathway participates in the pathophysiological process of diabetic myocardial fibrosis has not been reported. As shown in our results, both in vivo and in vitro, the protein expression of CaN and NFATc3 were both significantly elevated under hyperglycemic conditions. But treating NRCFs with CsA attenuated the increased protein expression of CaN, NFATc3 and fibrosis-related proteins. The immunofluorescence staining of NFATc3 showed that the distribution of NFATc3 increased significantly in the nucleus after treatment with high glucose, and this increase was attenuated after administration of CsA.

EZH2 is a catalytic subunit of polycomb repressive complex 2 and has been reported to have a role in podocytes injuries and oxidative stress in diabetes [26]. It is also closely related to impaired cardiac function and increased cardiomyocyte apoptosis [12]. In addition, Song et al. [11] found that EZH2 can regulate fibroblast differentiation through the Ang II-TGF-β1-Smad pathway, which subsequently promotes the occurrence of atrial fibrosis and atrial fibrillation. Meanwhile, previous study revealed that addition of CsA in T cells, to impair the translocation of NFAT to the nucleus, decreased the binding of EZH2 and the promoter of downstream protein, suggest that NFAT could interact with EZH2 to mediate gene transcription [27]. However, whether EZH2 is involved in diabetic ventricular myocardial fibrosis and whether there is a regulatory relationship between CaN/NFATc3 and EZH2 has not been elucidated. The results of the present study showed that diabetes upregulated the expression of EZH2, and their downstream expression of H3K27me3. while the increased expression of EZH2 and H3K27me3 was eliminated after treatment with CsA. Besides, GSK 126 treatment diminished the upregulated expression of fibrosis-related proteins in NRCFs cultured with high glucose. This was consistent with a recent study, in which GSK126 could reverse ISO-induced myocardial fibrosis [28]. In summary, our research demonstrated that CaN/NFATc3 played a crucial role in the process of myocardial fibrosis induced by diabetes. This pathway may regulate the expression of EZH2, which performed its classical function, leading to the upregulation of H3K27me3. However, further investigations are needed to find target genes for H3K27me3.

## Conclusion

In conclusion, the present study demonstrated that diabetes promotes myocardial fibrosis by activating the CaN/NFATc3/EZH2 pathway. Thus, targeting this pathway may be a potential treatment against DM-related myocardial fibrosis.

### Electronic supplementary material

Below is the link to the electronic supplementary material.

Supplementary Material 1

## Data Availability

The datasets generated and analysed during the current study are not publicly available due the principle of funding confidentiality but are available from the corresponding author upon reasonable request.

## Abbreviations

CaN: Calcineurin
NFATc3: Nuclear factor of activated T cells 3
EZH2: Enhancer of zeste homolog 2
H3K27me3: Trimethylates lysine 27 on histone 3
STZ: Streptozotocin
DM: Diabetes mellitus
Con: Controlled glucose
SD: Sprague-Dawley
EF: Ejection fraction
FS: Fractional shortening
LVIDd: Left ventricular internal diameter at end-diastole
LVIDs: Left ventricular internal diameter at end-systole
HE: Hematoxylin and eosin
NRCFs: Neonatal rat cardiac fibroblasts
PBS: Phosphate buffered saline
FBS: Fetal bovine serum
HG: High glucose

## References

[1] Nathan DM, Lachin JM, Bebu I (2022). Glycemia Reduction in Type 2 Diabetes - Microvascular and Cardiovascular Outcomes. N Engl J Med.

[2] Nakamura K, Miyoshi T, Yoshida M (2022). Pathophysiology and treatment of Diabetic Cardiomyopathy and Heart failure in patients with diabetes Mellitus. Int J Mol Sci.

[3] Salvador DB, Gamba MR, Gonzalez-Jaramillo N (2022). Diabetes and myocardial fibrosis: a systematic review and Meta-analysis. JACC Cardiovasc Imaging.

[4] Park HS, Lee SC, Cardenas ME (2019). Calcium-calmodulin-calcineurin signaling: a globally conserved Virulence Cascade in eukaryotic microbial pathogens. Cell Host Microbe.

[5] Liu X, Guo JW, Lin XC (2021). Macrophage NFATc3 prevents foam cell formation and atherosclerosis: evidence and mechanisms. Eur Heart J.

[6] Yan J, Honglei Y, Yun W (2022). Puerarin ameliorates myocardial remodeling of spontaneously hypertensive rats through inhibiting TRPC6-CaN-NFATc3 pathway. Eur J Pharmacol.

[7] Zhou H, Xia C, Yang Y (2022). The Prevention Role of Theaflavin-3,3’-digallate in Angiotensin II Induced Pathological Cardiac Hypertrophy via CaN-NFAT Signal Pathway. Nutrients.

[8] Asadi F, Razmi A, Dehpour AR (2016). Tropisetron inhibits high glucose-induced calcineurin/NFAT hypertrophic pathway in H9c2 myocardial cells. J Pharm Pharmacol.

[9] Cheng KC, Chang WT, Kuo FY (2019). TGR5 activation ameliorates hyperglycemia-induced cardiac hypertrophy in H9c2 cells. Sci Rep.

[10] Duan R, Du W, Guo W (2020). EZH2: a novel target for cancer treatment. J Hematol Oncol.

[11] Song S, Zhang R, Mo B (2019). EZH2 as a novel therapeutic target for atrial fibrosis and atrial fibrillation. J Mol Cell Cardiol.

[12] Wang C, Liu G, Yang H (2021). MALAT1-mediated recruitment of the histone methyltransferase EZH2 to the microRNA-22 promoter leads to cardiomyocyte apoptosis in diabetic cardiomyopathy. Sci Total Environ.

[13] Khodeer S, Era T (2017). Identifying the biphasic role of Calcineurin/NFAT signaling enables replacement of Sox2 in somatic cell reprogramming. Stem Cells.

[14] Zhang ZY, Dang SP, Li SS (2022). Glucose fluctuations aggravate myocardial fibrosis via the Nuclear factor-kappab-mediated nucleotide-binding oligomerization domain-like receptor protein 3 Inflammasome activation. Front Cardiovasc Med.

[15] Zhang L, Liu L, Li X (2020). TRAP1 attenuates H9C2 myocardial cell injury induced by extracellular acidification via the inhibition of MPTP opening. Int J Mol Med.

[16] Deng B, Zhang Y, Zhu C (2023). Divergent actions of myofibroblast and myocyte β-Adrenoceptor in Heart failure and fibrotic remodeling. Circul Res.

[17] Lee H, Park C, Lee S (2023). Systemic proinflammatory-profibrotic response in aortic stenosis patients with diabetes and its relationship with myocardial remodeling and clinical outcome. Cardiovasc Diabetol.

[18] Xie S, Zhang M, Shi W (2022). Long-term activation of glucagon-like peptide-1 receptor by Dulaglutide prevents Diabetic Heart failure and metabolic remodeling in type 2 diabetes. J Am Heart Association.

[19] Cosentino F, Grant P, Aboyans V (2020). 2019 ESC Guidelines on diabetes, pre-diabetes, and cardiovascular diseases developed in collaboration with the EASD. Eur Heart J.

[20] Yu H, Sun T, He X (2022). Association between Parkinson’s Disease and Diabetes Mellitus: from Epidemiology, Pathophysiology and Prevention to Treatment. Aging and Disease.

[21] Ho KL, Karwi QG, Connolly D (2022). Metabolic, structural and biochemical changes in diabetes and the development of heart failure. Diabetologia.

[22] Marino F, Salerno N, Scalise M (2023). Streptozotocin-Induced type 1 and 2 diabetes Mellitus Mouse Models Show different functional, Cellular and molecular patterns of Diabetic Cardiomyopathy. Int J Mol Sci.

[23] Hong M, Na S, Jang Y (2021). Betulinic Acid improves Cardiac-Renal Dysfunction caused by hypertrophy through Calcineurin-NFATc3 signaling. Nutrients.

[24] Zheng J, Tian J, Wang S (2020). Stachydrine hydrochloride suppresses phenylephrine-induced pathological cardiac hypertrophy by inhibiting the calcineurin/nuclear factor of activated T-cell signalling pathway. Eur J Pharmacol.

[25] Zhao Y, Cui G, Zhou N (2016). Calpain-calcineurin-nuclear factor signaling and the development of Atrial Fibrillation in patients with Valvular Heart Disease and Diabetes. J Diabetes Res.

[26] Wan J, Hou X, Zhou Z (2017). WT1 ameliorates podocyte injury via repression of EZH2/β-catenin pathway in diabetic nephropathy. Free Radic Biol Med.

[27] Hod-Dvorai R, Jacob E, Boyko Y (2011). The binding activity of Mel-18 at the Il17a promoter is regulated by the integrated signals of the TCR and polarizing cytokines. Eur J Immunol.

[28] Aziz S, Yalan L, Raza MA (2023). GSK126 an inhibitor of epigenetic regulator EZH2 suppresses cardiac fibrosis by regulating the EZH2-PAX6-CXCL10 pathway. Biochem Cell Biol.

