# Supplementary material for: Calcineurin/NFATc3 pathway mediates myocardial fibrosis in diabetes by impairing enhancer of zeste homolog 2 of cardiac fibroblasts
Source: BMC Cardiovasc Disord. 2023 Sep 21;23:474. doi: 10.1186/s12872-023-03492-5 (PMC10512648; doi:10.1186/s12872-023-03492-5)

Original blots

Red boxes represent the location of target proteins;

Green boxes represent the representative blots used in the manuscript.

Figure 2B Collagen I + TGF-β1 + β-actin

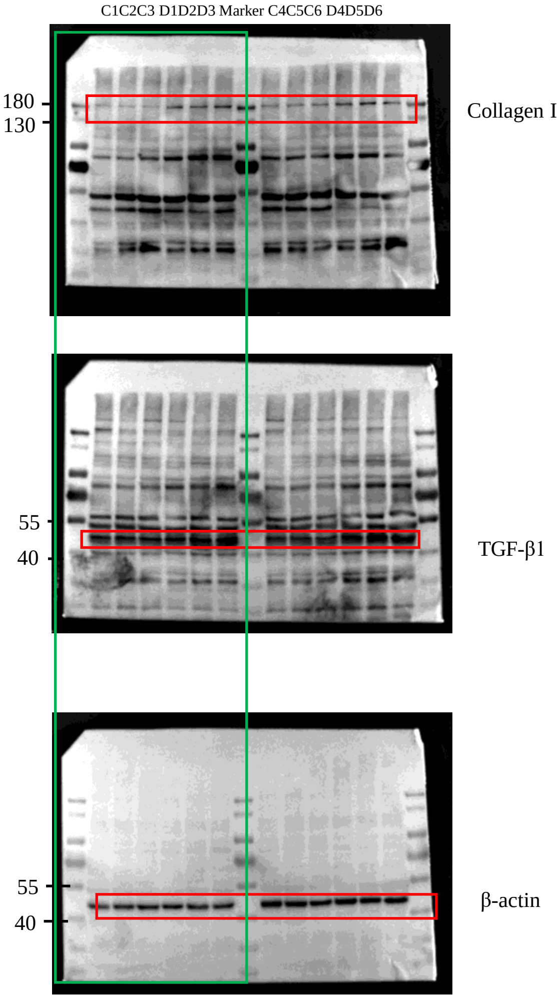

Figure 2C Collagen I + β-actin

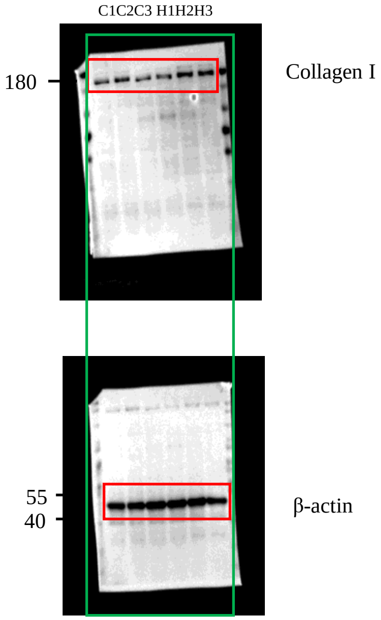

Figure 2C TGF-β1 + β-actin

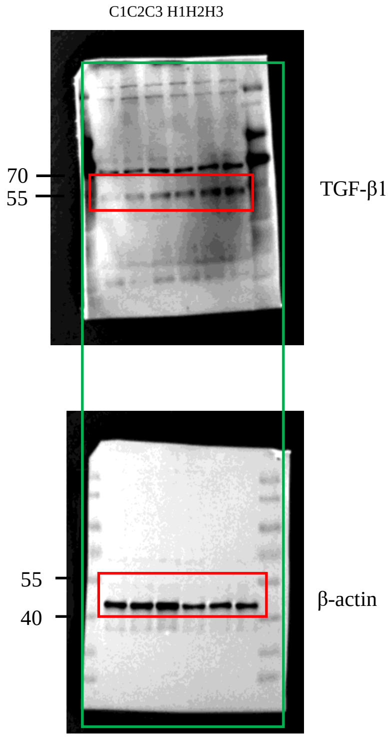

Figure 3A CaN + β-actin

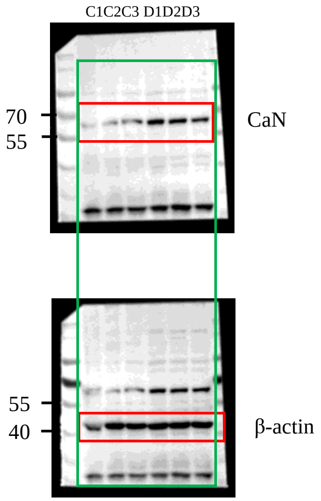

Figure 3A NFATc3 + β-actin

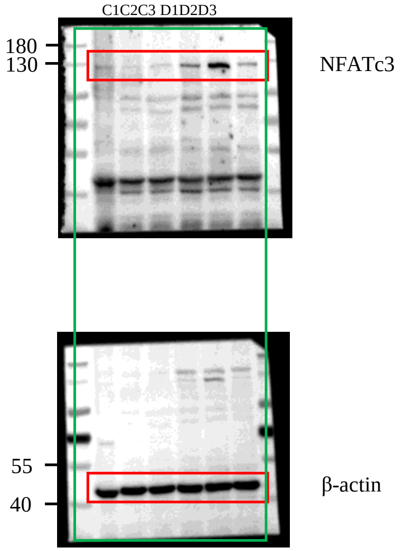

Figure 3A EZH2 + β-actin

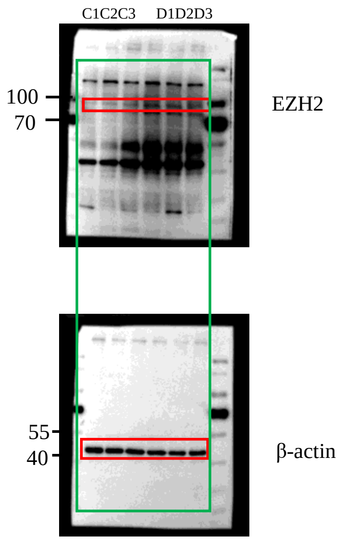

Figure 3A H3K27me3 + H3

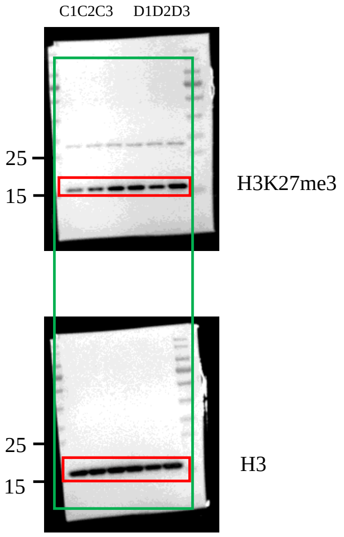

Figure 3F CaN + β-actin

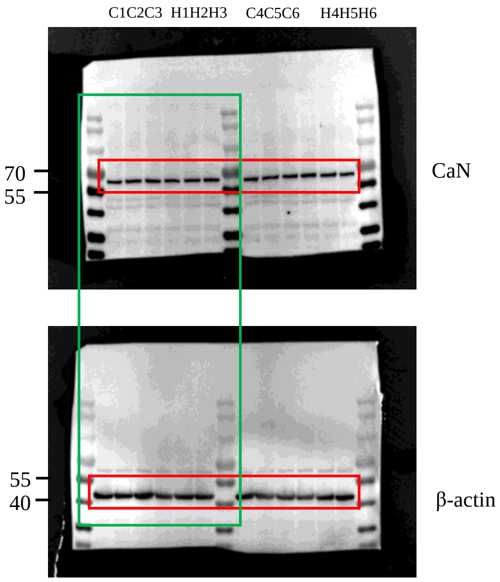

Figure 3F NFATc3 + β-actin

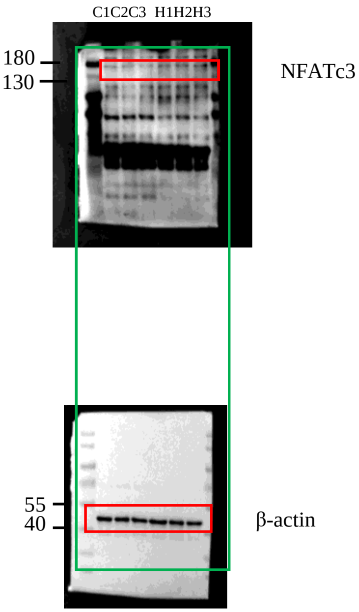

Figure 3F EZH2 + β-actin

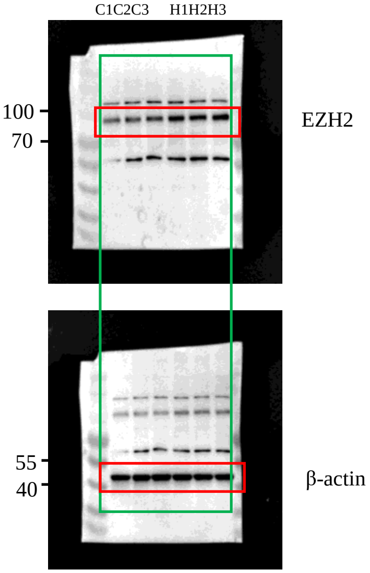

Figure 3F H3K27me3 + H3

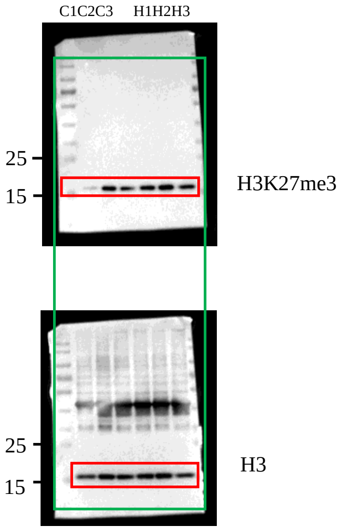

Figure 4 Collagen I + β-actin

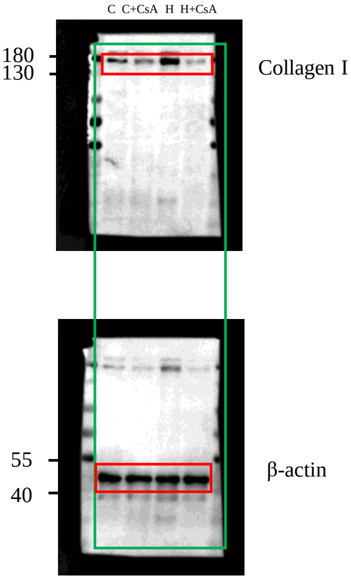

Figure 4 Collagen III + TGF-β1 + β-actin

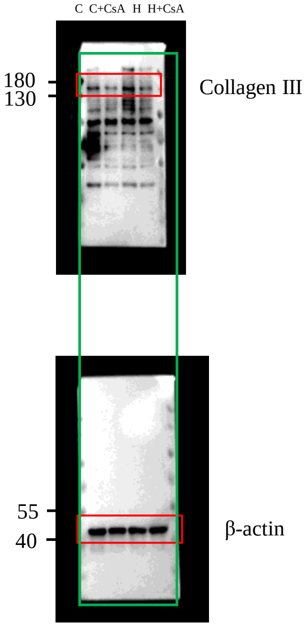

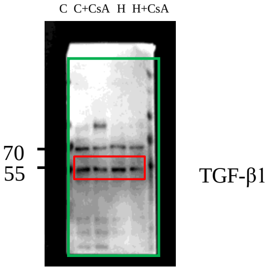

Figure 5 CaN + β-actin

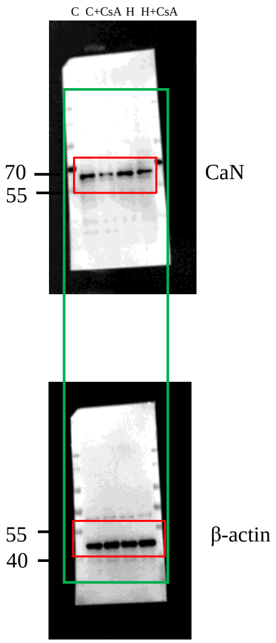

Figure 5 NFATc3 + β-actin

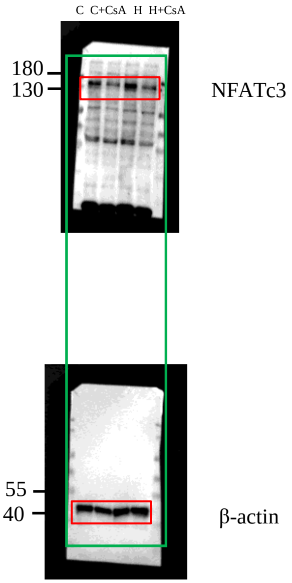

Figure 5 EZH2 + β-actin

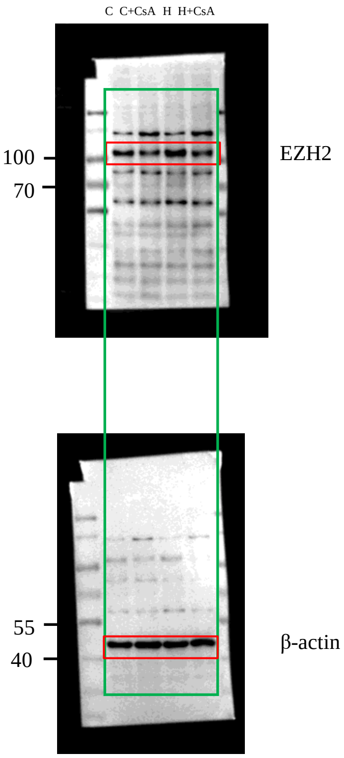

Figure 5 H3K27me3 + H3

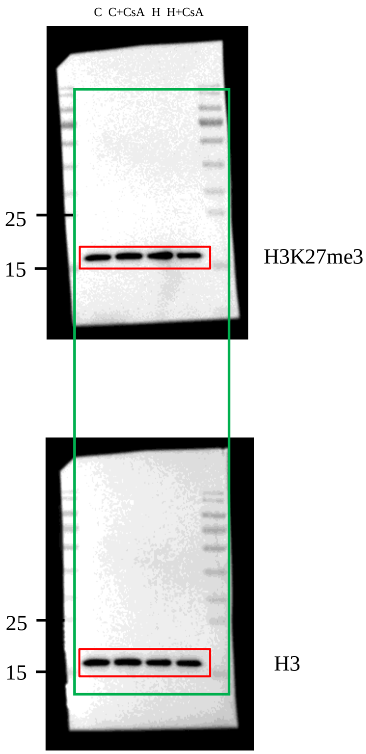

Figure 6 Collagen I + β-actin

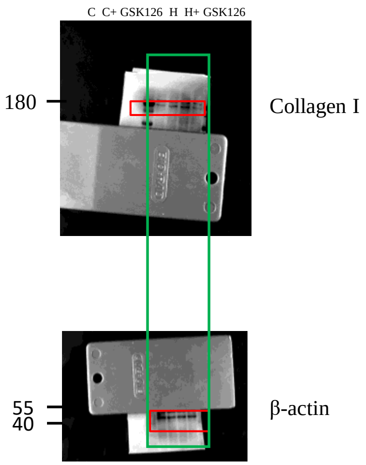

Figure 6 Collagen III + β-actin

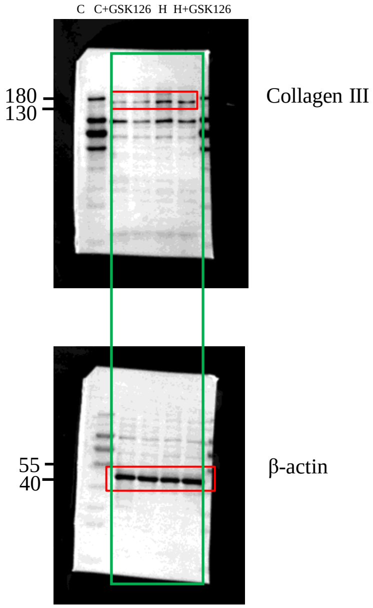

Figure 6 TGF-β1+ β-actin

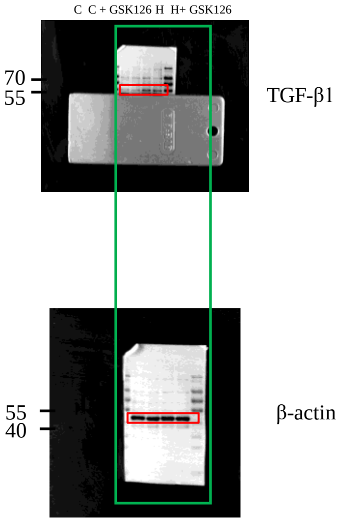

Supplement: Supplementary file 1 — Supplementary Material 1 [file 12872_2023_3492_MOESM1_ESM.pdf]
